# Effects of Soccer Heading on Brain Structure and Function

**DOI:** 10.3389/fneur.2016.00038

**Published:** 2016-03-21

**Authors:** Ana Carolina Rodrigues, Rodrigo Pace Lasmar, Paulo Caramelli

**Affiliations:** ^1^Pró-Reitoria de Graduação, Reitoria da Universidade Federal de Minas Gerais, Belo Horizonte, Minas Gerais, Brazil; ^2^Grupo de Pesquisa em Neurologia Cognitiva e do Comportamento, Departamento de Clínica Médica, Faculdade de Medicina da Universidade Federal de Minas Gerais, Belo Horizonte, Minas Gerais, Brazil; ^3^Faculdade de Ciências Médicas de Minas Gerais, Belo Horizonte, Minas Gerais, Brazil

**Keywords:** sports, soccer, heading, injury, brain, cognition

## Abstract

Soccer is the most popular sport in the world, with more than 265 million players worldwide, including professional and amateur ones. Soccer is unique in comparison to other sports, as it is the only sport in which participants purposely use their head to hit the ball. Heading is considered as an offensive or defensive move whereby the player’s unprotected head is used to deliberately impact the ball and direct it during play. A soccer player can be subjected to an average of 6–12 incidents of heading the ball per competitive game, where the ball reaches high velocities. Moreover, in practice sessions, heading training, which involves heading the ball repeatedly at low velocities, is common. Although the scientific community, as well as the media, has focused on the effects of concussions in contact sports, the role of subconcussive impacts, as it can occur during heading, has recently gained attention, considering that it may represent an additional mechanism of cumulative brain injury. The purpose of this study is to review the existing literature regarding the effects of soccer heading on brain structure and function. Only in the last years, some investigations have addressed the impact of heading on brain structure, by using neuroimaging techniques. Similarly, there have been some recent studies investigating biochemical markers of brain injury in soccer players. There is evidence of association between heading and abnormal brain structure, but the data are still preliminary. Also, some studies have suggested that subconcussive head impacts, as heading, could cause cognitive impairment, whereas others have not corroborated this finding. Questions persist as to whether or not heading is deleterious to cognitive functioning. Further studies, especially with longitudinal designs, are needed to clarify the clinical significance of heading as a cause of brain injury and to identify risk factors. Such investigations might contribute to the establishment of safety guidelines that could help to minimize the risk of possible adverse effects of soccer on brain structure and function.

## Introduction

Soccer is the most popular sport in the world, with more than 265 million players worldwide, including professional and amateur ones (1). Although it is a sport not traditionally identified as high-risk for concussions (2–4), soccer players are prone to traumatic brain injury (5–7), and up to 22% of all soccer injuries are concussions (8). Furthermore, several studies have shown that concussion rates in soccer are comparable to, and often exceed, those of other contact sports traditionally considered as inherently more violent, such as football and ice hockey (9). A prospective study investigating female middle-school soccer players (10) reported that heading the ball accounted for 30.5% of concussions. Similarly, a recent retrospective analysis involving high-school soccer players (11) showed that heading was responsible for 30.6% of concussions among boys and 25.3% among girls, although the most frequent mechanism of injury in heading-related concussions was player–player contact during the ball dispute.

Head injury during soccer is usually the result of either direct contact (e.g., head vs. head, head vs. knee, and head vs. the ground) or contact with the ball while heading it. In this sense, soccer is unique in comparison to other sports, as it is the only sport in which participants purposely use their head to hit the ball. Heading is considered an offensive or defensive move whereby the player’s unprotected head is used to deliberately impact the ball and direct it during play. Players may head the ball to pass to another player, move the ball down the field, or score a goal. To counter to external forces to the head during impact, players must prepare for impact by bracing the neck musculature and properly execute the technique by moving the entire body in one motion (12). A soccer player can be subjected to an average of 6–12 incidents of heading the ball per competitive game, where the ball reaches high velocities (13). Moreover, in practice sessions, heading training, which involves heading the ball repeatedly at low velocities, is common. Although the scientific community, as well as the media, has focused on the effects of concussions in contact sports, the role of subconcussive impacts, as it can occur during heading, has recently gained attention, considering that it may represent an additional mechanism of cumulative brain injury. The term “subconcussive” was proposed to describe the impact to the head that may cause neuronal dysfunction in the absence of concussive symptoms (14).

Heading involves repeated impact, acceleration–deceleration of the brain inside the skull, and possibly rotation of the brain. Moreover, cumulative effects of repetitive minor injury may not manifest for many years, as in chronic traumatic encephalopathy (15). Therefore, pathological evidence of traumatic brain injury, if detectable, is likely to present prior to onset of overt symptoms or disability. Importantly, the possible negative effects of heading may depend on the rate of exposures, the time between exposures, and the vulnerability of individual players (15).

The purpose of this study is to review the existing literature regarding the effects of soccer heading on brain structure and function. These investigations have explored the consequences of immediate (e.g., after a soccer match), short-term (e.g., after one or few soccer seasons), and long-term (e.g., after many soccer seasons) heading exposure. Only in the last years, some studies have addressed the impact of heading on brain structure, by using neuroimaging techniques. Similarly, there have been some recent studies investigating biochemical markers of brain injury in soccer players. There is evidence of association between heading and abnormal brain structure, but the data are still preliminary. Also, some studies have suggested that subconcussive head impacts, as heading, could cause cognitive impairment, whereas others have not corroborated this finding.

We searched three databases – such as PubMed, LILACS, and Scopus – for articles published until December 2015, by using the terms “soccer,” “heading,” and “brain.” The search retrieved 92 articles published from 1981 to 2015. Considering the inclusion criteria, which involved papers with original research design, English language, and focus on the effects of soccer heading on brain structure and/or function, 29 articles were selected for the present review. We had full access to all these studies, except to two of them.

## Effects of Soccer Heading on Brain Structure

Some investigations have demonstrated changes in brain structure of soccer players and suggested an association between these changes and soccer heading. However, the data are still preliminary, as summarized in Table 1.

**Table 1:** **Summary of the studies investigating the effects of soccer heading on brain structure**.

Reference	Samples	Methods	Heading exposure	Main results
**Neuroimaging**				
Sortland and Tysvaer (16)	Male former professional soccer players	CCT	Long-term exposure	One-third of the players had slight to moderate central atrophy with widening of the lateral ventricles
Lipton et al. (15)	Male and female amateur soccer players	Diffusion tensor MRI	Short-term exposure	High frequency of heading was associated with lower FA at three locations in temporo-occipital white matter
Koerte et al. (18)	Male former professional soccer players and non-contact sport athletes	MRI	Long-term exposure	Greater cortical thinning with increasing age in the right inferolateral–parietal, temporal, and occipital cortex was demonstrated in soccer players compared to controls
Jordan et al. (21)	Male professional soccer players and track athletes	MRI	Long-term exposure	No differences were verified in brain structure between soccer players and controls
**Biochemical markers of brain injury**				
Mussack et al. (22)	Male amateur soccer players and patients after minor traumatic brain injury	Analysis of serum S-100B levels	Immediate exposure	S-100B serum levels were elevated after heading when compared to normal exercise. None of the soccer players reached S-100B serum levels verified in subjects showing traumatic brain injury
Stålnacke et al. (24)/Stålnacke et al. (25)	Male professional soccer players/female professional soccer players	Analysis of serum S-100B and NSE levels	Immediate exposure	Serum levels of S-100B and NSE increased after a game. Increases in S-100B were positively correlated to the number of headings and of other trauma events
Straume-Naesheim et al. (26)	Male professional soccer players	Analysis of serum S-100B levels	Immediate exposure	Serum levels of S-100B increased after a regular league match, with or without head impact, after a high-intensity training session without heading, and after a low-intensity training session with heading exercises. The increase for the match groups was higher than for the training groups, but no differences were seen between the two match groups or the two training groups
Bamaç et al. (27)	Male professional soccer players	Analysis of serum NGF and BDNF levels	Immediate exposure	Serum levels of NGF and BDNF were elevated in response to heading exercises
Koerte et al. (29)	Male former professional soccer players and non-contact sport athletes	Magnetic resonance spectroscopy	Long-term exposure	Increases in choline and myo-inositol were verified in soccer players when compared with controls. Myo-inositol and glutathione were positively correlated with lifetime estimate of headings
Zetterberg et al. (30)	Male amateur soccer players and non-athletic subjects	Analysis of serum and/or cerebrospinal fluid concentrations of NF-L, T-tau, GFAP, S-100B, and albumin	Immediate exposure	There were no differences in concentrations of biomarkers of brain injury between soccer players who performed 10 or 20 headings or between either of these two groups and the control group. Biomarker levels did not correlate with the number of headings
Stålnacke and Sojka (31)	Male amateur soccer players	Analysis of serum S-100B levels	Immediate exposure	There were no increases in serum levels of S-100B after a heading session. No differences were seen in S-100B between players who performed or not headings, either before or after the session

*CCT, cerebral computed tomography; MRI, magnetic resonance imaging; FA, fractional anisotropy; NSE, neuron-specific enolase; NGF, nerve growth factor; BDNF, brain-derived neurotrophic factor; NF-L, neurofilament light protein; T-tau, total tau protein; GFAP, glial fibrillary acidic protein*.

### Neuroimaging

A small number of studies have investigated the impact of heading on brain structure by using neuroimaging techniques. An early study by Sortland and Tysvaer (16) evaluated male former professional soccer players, aged between 39 and 68 years, who were submitted to cerebral computer tomography (CCT) with assessment for brain atrophy, visually and by linear measurements. The results showed that, by visual grading, about one-third of the players had slight to moderate central atrophy with widening of the lateral ventricles, which was strongly supported by linear measurements compared to normal controls. The authors assumed that the brain damage is a result of playing soccer for years, therefore a consequence of long-term heading exposure, with multiple small head injuries mainly connected with heading.

More recently, Lipton et al. (15) investigated white matter microstructure in male and female amateur soccer players, with a mean age of 30.9 years, by using the diffusion tensor magnetic resonance imaging (MRI) technique. The primary imaging outcome was fractional anisotropy (FA), which provides assessment of the degree of anisotropic diffusion, defined as the presence of diffusional movements in different directions, occurring within a brain region. FA tends to be high in regions of high cellular organization, and low in regions where the cells are not specifically oriented (17). Therefore, FA is often used to measure the integrity of white matter, since it reflects the degree of myelination and axonal density. The participants completed a questionnaire to quantify heading in the prior 12 months, which characterizes a short-term heading exposure, and were also submitted to a computerized neuropsychological evaluation, which aimed to assess psychomotor speed, attention, executive function, and memory. Each soccer player headed approximately 432 times over the previous year. High frequency of heading was associated with lower FA at three locations in temporo-occipital white matter, and also with poorer performance in the memory test. Interestingly, these associations were not linear, but there were thresholds in terms of number of headings needed to trigger FA reduction (between 885 and 1550 headings per year, depending on the brain region) and cognitive impairment (1800 headings per year). The results, which suggest an association between heading and abnormal white matter microstructure and also poorer cognitive performance, were not explained by lifetime concussion history and demographic features. Nonetheless, a limitation of this study is its cross-sectional nature, which avoids the establishment of a clear causal relationship between heading and brain changes.

Koerte et al. (18), in a recent investigation, evaluated cortical thickness in male former professional soccer players, with a mean age of 49.3 years, compared to age- and gender-matched former professional non-contact sport athletes, by using high-resolution structural MRI. All individuals, in both groups, were still actively participating in their respective sports at the time of the study. Soccer players were asked to inform how many headings they performed per week during the past 12 months prior to the investigation, and such a number was multiplied by the total years of formal training in soccer in order to obtain a lifetime estimate of headings, that is, an approximate calculation of long-term heading exposure. The results demonstrated greater cortical thinning with increasing age in the right inferolateral–parietal, temporal, and occipital cortex in soccer players compared to controls. In addition, cortical thinning in soccer players was associated with lower cognitive processing speed in the *Trail Making Test A* (19), which measures visual search and psychomotor speed, as well as with estimated exposure to repetitive subconcussive head impact. Also, a cognitive comparison between groups revealed decreased memory performance in soccer players, in relation to controls, in the *Rey–Osterrieth Complex Figures Test* (20), which measures visuoconstruction, planning and organization, and visual memory, although findings were in the normal range for both groups. According to the researchers, despite the limitations of the study, which include the small sample size and athletes self-report about history of headings, the results suggest that repetitive subconcussive head impact may play a role in age-related cortical thinning that may lead to early cognitive decline in soccer players.

On the other hand, a previous study by Jordan et al. (21) failed to find any evidence of brain structure damage in soccer players. Male professional soccer players, with a mean age of 24.8 years, were compared with age- and gender-matched elite track athletes with respect to a questionnaire regarding symptoms of head and neck injuries, as well as to MRI abnormalities. A heading exposure index was developed to assess a dose–response effect of chronic heading over the player’s career. Thus, the authors aimed to examine the consequences of long-term heading exposure. Questionnaire analysis and MRI results demonstrated no significant differences between groups. Among the soccer players, there was no correlation between the outcome variables and heading exposure parameters. However, reported head injury symptoms correlated significantly with histories of prior acute head injuries. According to the researchers, these findings suggest that any evidence of brain trauma in soccer players relates more to acute head injuries than repetitive heading.

### Biochemical Markers of Brain Injury

Some studies have specifically investigated biochemical markers of brain damage in soccer players, as well as their relationship with head impacts. Mussack et al. (22) measured serum levels of S-100B, a calcium-binding protein that is present in the astroglial cells of the central nervous system, in male young amateur soccer players, aged between 12 and 17 years, before and after controlled heading and normal exercise, as well as in older patients after minor traumatic brain injury. Previous studies have demonstrated that increased serum concentrations of this biomarker may reflect the presence and severity of brain tissue damage (23). The results of this investigation, which explored the effects of immediate heading exposure, demonstrated that the increases in S-100B serum levels 1 h after a session of heading and also after a session of normal exercise were insignificant and that these concentrations returned to the starting values 6 h after both training sessions. However, S-100B serum levels were significantly elevated, at the three measurement points, after heading when compared to normal exercise. Importantly, none of the young amateur soccer players reached S-100B serum levels verified in subjects showing traumatic brain injury with visible brain damage.

Stålnacke et al. (24) analyzed serum concentrations of two biochemical markers – S-100B and neuron-specific enolase (NSE), a cytoplasmatic enzyme that occurs predominantly in neurons and is also considered as a biomarker of brain tissue damage (23) – in male professional soccer players with a mean age of 26 years. Blood samples were taken from the participants before and after a competitive game and the numbers of headings and of trauma events during soccer play were obtained by video recordings. The results showed that serum concentrations of both S-100B and NSE significantly increased after the game in comparison with the pre-game values. Also, increases in S-100B were significantly and positively correlated to the number of headings and to the number of other trauma events. However, as emphasized by the authors, although heading may have contributed to these increases, the mechanisms involved in the rise of serum concentrations of the biochemical markers are not known yet. A further study by Stålnacke et al. (25), which also examined the consequences of immediate heading exposure, aimed to investigate serum levels of S-100B and NSE in female professional soccer players, with a mean age of 23 years. The results were very similar to that verified in male players, that is, the game induced increases in serum concentrations of S-100B and NSE, and there were significant correlations between the number of headings and of other trauma events and S-100B level increase.

In a study involving male professional soccer players aged between 19 and 35 years, Straume-Naesheim et al. (26) explored the effects of immediate heading exposure, by comparing serum levels of S-100B under four different conditions: after a head impact occurring during a regular league match, after a regular league match with no recorded head trauma, after a high-intensity training session without heading, and after a low-intensity training session with heading exercises only. Blood samples were taken at baseline, within 1 h after the match or the training session (B1), and the next morning (B12). All groups had a significant increase in serum concentrations of S-100B between baseline and B1 and a similar significant decrease from B1 to B12. The increase for the match groups was significantly higher than for the training groups, but no significant differences were seen between the two match groups or the two training groups for any of the sampling time points. In the match group without head trauma, there was no correlation between serum level of S-100B and number of headings. Furthermore, in the training group with heading exercises, no relationship was detected between serum concentration of S-100B and perceived heading intensity. The results suggest that both soccer matches and soccer training cause a transient increase in S-100B. According to the researchers, there is a possible additive effect of high-intensity exercise and heading, but minor head impacts do not seem to cause an additional increase.

Bamaç et al. (27) investigated the effects of immediate heading exposure on serum levels of nerve growth factor (NGF) and brain-derived neurotrophic factor (BDNF) in male professional soccer players with a mean age of 24 years. NGF and BDNF, members of the neurotrophin family, are argued to be reliable markers of brain damage when released into the circulation (28). Each player completed a series of 15 headings and blood samples were obtained just before and after the training. The results showed that NGF and BDNF serum levels were significantly elevated in response to heading exercise. The authors speculate that the microtrauma caused by repetitive heading and/or the course of survival of the injured neurons may lead to increased NGF and BDNF levels. However, they acknowledge that measurements in peripheral blood can reflect only a limited view of the whole metabolism of these neurotrophins.

Another recent study by Koerte et al. (29) evaluated brain neurochemistry, by using magnetic resonance spectroscopy, in male former professional soccer players, with a mean age of 52 years, without a known history of concussion, in comparison to age- and gender-matched former professional non-contact sport athletes. As in their more recent investigation (18), which involved nearly the same groups of subjects, all participants were still actively participating in their respective sports at the time of the study and, in the group of soccer players, lifetime estimate of headings, an approximate calculation of long-term heading exposure, was also based on the athlete’s self-report. The results showed significant increases in choline, a marker of membrane disruption, and myo-inositol, a marker of glial activation, in soccer players when compared with controls. In addition, myo-inositol and glutathione, an anti-oxidant, were significantly and positively correlated with lifetime estimate of headings in the soccer group. A brief cognitive and balance evaluation revealed no significant differences between groups. This study suggests, as pointed out by the authors, a possible association between heading and altered brain neurochemistry in soccer players. It is possible that even subconcussive head impacts may affect brain chemical concentrations and precede cognitive alterations, although these data are still preliminary.

It is important to emphasize that research on biochemical markers of brain damage in soccer players has also produced controversial findings. Zetterberg et al. (30), who aimed to investigate the consequences of immediate heading exposure, examined serum and cerebrospinal fluid concentrations of some biomarkers of brain injury in male amateur soccer players aged between 19 and 32 years and age- and gender-matched non-athletic control subjects. The players participated in a training session that involved heading a ball kicked from a distance of 30 m. Some players were instructed to perform 10 headings, whereas others were required to perform 20 headings. The participants underwent lumbar puncture – for measurement of neurofilament light protein (NF-L), total tau protein (T-tau), glial fibrillary acidic protein (GFAP), S-100B, and albumin – and serum sampling – for measurement of S-100B and albumin – 7–10 days after the training session. There were no significant differences in serum and cerebrospinal fluid concentrations between soccer players who had performed 10 or 20 headings or between either of these two groups and the control group. Also, biomarker levels did not correlate with the number of headings performed. Therefore, the results suggest that standardized headings in soccer are not associated with known biochemical signs of acute brain injury.

Another study, conducted by Stålnacke and Sojka (31), also explored the effects of immediate heading exposure and aimed to analyze whether the controlled heading of soccer balls was associated with increased serum concentrations of S-100B. Male amateur soccer players, with a mean age of 22 years, were randomly divided into two groups. Players in the experimental group were instructed to perform 5 headings of a ball which was dropped from a height of 18 m, while players in the control group performed no headings. Blood samples were taken before and 30 min, 2, and 4 h after the heading session. The results showed no significant increases in serum concentrations of S-100B in the experimental group at any time point after headings, in comparison with baseline measures. Also, there were no significant differences in serum levels of S-100B between groups, either before or after the heading session. The researchers argue that, in this investigation, the impact probably was not sufficient to cause biochemically discernible damage of brain tissue.

## Effects of Soccer Heading on Brain Function

Similar to the research addressing the effects of soccer heading on brain structure, the literature investigating the effects of these subconcussive impacts on brain function has produced contradictory findings, as summarized in Tables 2 and 3. Tysvaer and Løchen (32) examined the consequences of long-term heading exposure, by investigating neuropsychological performance of male former professional soccer players aged between 35 and 64 years. The players were compared with a control group of age- and education-matched hospitalized patients suffering from a variety of disorders, but having no history of head or neck injuries and no evidence of brain damage. The neuropsychological examination, which included tests of attention, concentration, memory, and judgment, demonstrated that 81% of the soccer players had some degree of impairment, compared to 40% of the controls with only a mild degree of impairment. The authors hypothesize that their findings probably reflect the cumulative result of repeated traumas from heading the ball. However, the study offers no evidence of a clear association between heading and cognitive impairment.

**Table 2:** **Summary of the studies with evidence of brain function impairment in soccer players**.

Reference	Samples	Methods	Heading exposure	Main results
Tysvaer and Løchen (32)	Male former professional soccer players and patients with no evidence of brain injury	Extensive neuropsychological test battery	Long-term exposure	81% of the soccer players had mild-to-severe deficits in tests of attention, concentration, memory, and judgment, compared to 40% of the controls with a mild degree of impairment
Matser et al. (7)	Male professional soccer players and non-contact sport athletes	Extensive neuropsychological test battery	Short-term exposure	Soccer players had poorer performance on verbal and visual memory, planning, and visuoperceptual processing tasks, compared with controls. An increasing number of headings and concussions were associated negatively with cognitive functioning. Forward and defensive players performed poorer than midfield players and goalkeepers on some tests
Downs and Abwender (33)	Male and female amateur and professional soccer players and swimmers	Four tests measuring motor speed, attention, concentration, reaction time, and conceptual thinking	Long-term exposure	Soccer players performed worse than swimmers on conceptual thinking. Older players performed poorly than all other subgroups on conceptual thinking and reaction time. Estimates of heading predicted poorer performance on conceptual thinking
Webbe and Ochs (34)	Male amateur and professional soccer players	Extensive neuropsychological test battery	Immediate and long-term exposure	Soccer players with the highest self-reported estimates of heading who experienced headings within the previous 7 days scored lower on tests of verbal learning, verbally based conceptual performance, planning and attention, and information processing speed than other combinations of heading and recency
Rutherford et al. (35)	Male amateur soccer players and rugby and non-contact sport players	Extensive neuropsychological test battery	Long-term exposure	Performance of soccer players was worse than that of rugby and non-contact sport players on divided attention. Cumulative head injury and cumulative heading were marginal predictors of poorer performance on some tests
Zhang et al. (38)	Female amateur soccer players and non-soccer players	One test measuring executive functioning	Immediate exposure	Soccer players were slower than controls. There was an association between slower reaction times and increased hours of soccer per week and years of soccer experience

**Table 3:** **Summary of the studies without evidence of brain function impairment in soccer players**.

Reference	Samples	Methods	Heading exposure	Main results
Tysvaer and Storli (39)	Male professional soccer players	Questionnaire elaborated to record the incidence of head injuries due to heading	Long-term exposure	50% of the soccer players reported acute symptoms (e.g., disorientation), 16.4% related protracted symptoms (e.g., headache), and only 4.7% described prolonged symptoms (e.g., weakened memory) due to heading
Putukian et al. (40)	Male and female amateur soccer players	Four tests measuring reaction time, concentration, attention span, speed of information processing, divided attention, and active problem solving	Immediate exposure	There were no differences in pre-test or in post-test scores between athletes who participated in a session of heading practice and athletes who abstained from heading during exercises
Janda et al. (12)	Male and female amateur soccer players	Four tests measuring verbal learning, attention, tracking, information processing speed, and memory	Short-term exposure	After 1 year, no differences were found when comparing pre-season with post-season testing scores. There was no difference between the scores in this study and the standardized norms. There was no correlation between the number of ball impacts and cognitive performance, with the exception of a weak inverse association involving verbal learning in the second year
Stephens et al. (41)	Male amateur soccer players and rugby and non-contact sport players	Extensive neuropsychological test battery	Long-term exposure	There was no difference between groups in scores of all tests. There was no relationship between either cumulative head injury or cumulative heading and cognitive functioning. The only exception was a marginal prediction of poorer performance on divided attention by cumulative heading
Straume-Naesheim et al. (42)	Male professional soccer players	Extensive neuropsychological test battery	Long-term exposure	There was no association between estimated match or lifetime heading exposure and cognitive performance. Only 1.5% of the players qualified as outliers for one or more subtests when compared with the normal range
Kaminski et al. (44)	Female amateur high-school and college soccer players and non-athletes	Two tests measuring concentration, immediate memory recall and verbal memory	Short-term exposure	In both the college and high-school soccer groups, there were no correlations between the total number of headings and the change in scores of all outcome measures from pre-season to post-season. There were no differences between the three groups
Kaminski et al. (45)	Female amateur soccer players	Extensive neuropsychological test battery	Short-term exposure	There was no relationship between the number of headings and neuropsychological performance. None of the cognitive measures demonstrated decreases in performance over a soccer season
Kontos et al. (46)	Male and female amateur soccer players	Extensive neuropsychological test battery and symptom report	Short-term exposure	There were no differences in cognitive performance or symptoms among low, moderate, and high heading exposure groups
Vann Jones et al. (48)	Male retired professional soccer players	One test measuring memory	Long-term exposure	10.9% of the soccer players scored positively for possible mild cognitive impairment or dementia. There was no association between low-risk and high-risk playing positions, respectively, associated with reduced and greater frequency of heading, as well as length of playing career, and a positive screening result

A study conducted by Matser et al. (7) compared male professional soccer players (mean age 25.4 years) with a group of age- and gender-matched elite non-contact sport athletes for level of cognitive functioning, by using an extensive neuropsychological test battery. Professional soccer players reported a median of 800 headings during competitive matches in one soccer season, a period of time that can be considered as involving short-term heading exposure, and 54% of them experienced one or more soccer-associated concussions with or without loss of consciousness. The results showed that soccer players exhibited more cognitive impairment when compared with controls, as they had poorer performance on verbal and visual memory, planning, and visuoperceptual processing tasks. Moreover, an increasing number of headings and concussions incurred during soccer participation were associated negatively with cognitive functioning. Field position also influenced performance on neuropsychological testing, as forward and defensive players performed significantly poorer than midfield players and goalkeepers on some tasks. According to the researchers, the results suggest that participation in professional soccer may affect adversely some aspects of cognition, which appears to be attributed to increased frequency of heading the ball and soccer-related concussions, although more investigations are needed to allow extrapolating the findings to amateur or lower exposure soccer players.

Downs and Abwender (33) compared male and female soccer players with swimmers on neuropsychological tests assessing motor speed, attention, concentration, reaction time, and conceptual thinking. Each group of participants was composed of college and professional athletes, with a mean age of 19.6 and 42.1 years, respectively. The results showed that soccer players performed worse than swimmers on measures of conceptual thinking. In particular, the subgroup of older soccer players performed poorer than all other subgroups on measures of conceptual thinking and reaction time. The neuropsychological test scores did not vary as a function of reported history of concussion. Moreover, in the group of soccer players, estimates of career exposure to brain trauma, based on length of career and level of play, an approximate calculation of long-term heading exposure, predicted significantly poorer performance on measures of conceptual thinking, even after statistically controlling for age. As pointed out by the authors, these results suggest that playing soccer may be associated with cognitive impairment, although, due to the cross-sectional design of the investigation, heading may not be specifically implicated as a cause.

In a study which explored the effects of immediate heading exposure, by investigating the interaction between recent heading activity and current heading frequency, as well as the consequences of long-term heading exposure, Webbe and Ochs (34) evaluated the cognitive performance of male amateur and professional soccer players aged between 16 and 34 years. Participants were administered a battery of 6 neuropsychological tests and provided a report of their heading practices by answering a structured interview. Players with the highest self-reported estimates of current heading who also experienced headings within the previous 7 days scored significantly lower on tests that measured verbal learning, verbally based conceptual performance, planning and attention, and information processing speed than the other groups, characterized by heading frequency (high, moderate, or low) and recency (presence or absence of heading practice within the previous 7 days). On the other hand, the basic comparison of neuropsychological performance of soccer players vs. age- and gender-matched control athletes, as well as the comparison across the heading groups showed, at most, a weak heading effect. Also, no significant effect was found for estimates of lifetime heading on neuropsychological performance. The researchers argue that although it is not possible to isolate heading from other sources of head impacts, the results suggest that heading the ball may be a factor sufficient to depress, at least temporarily, cognitive functioning in some players.

Rutherford et al. (35) compared male university soccer players (mean age 20.5 years) with age- and gender-matched rugby and non-contact sport players, on a range of 16 neuropsychological tests. Cumulative head injury incidence and cumulative heading, an approximate calculation of long-term heading exposure, were estimated by using self-reports and a combination of observation and self-reports, respectively. The only significant difference between groups was in accuracy scores of *Test of Attention Performance (TAP) – Divided Attention* (36). After control for the influence of the number of head injuries sustained, performance of soccer players was significantly worse than that of rugby and non-contact sport players. Cumulative head injury was a marginal predictor of *Trail Making Test B*, which measures executive control, and *TAP – Divided Attention* latencies in a positive fashion. Also, cumulative heading was a marginal predictor of the number of category shifts in the *Wisconsin Card Sorting Test* (37), which measures executive functioning abilities, in a negative fashion. The authors emphasize that, as consequence of the exploratory analysis, it would be inappropriate to interpret their results as clear evidence of an association between soccer practice, including heading, and neuropsychological impairment. As they point out, this study is limited to identifying relations worthy of further confirmatory examination.

Zhang et al. (38) aimed to investigate if frequent head-to-ball contact could cause cognitive dysfunctions and brain injury to soccer players and compared two groups of high-school female students aged between 15 and 18 years – soccer and non-soccer players – by using a tablet-based approach designed to evaluate executive functioning. This study involved a task in which a visual target appeared randomly at one of the four locations in the screen. In the first situation, the participant was instructed to touch the response box containing the target and, in the second situation, the subject had to touch the response box opposite to the target location. Every soccer player performed head balls during the practice session before the testing, with median 6 head balls per session based on self-reports. No participant in the non-soccer group performed a head ball before testing. Therefore, the authors explored the consequences of immediate heading exposure. Although there were no differences between groups in reaction times in the first situation, soccer players were slower than controls in the second situation, which indicates a disruption specific to voluntary responses. Also, the data showed an association between slower reaction times and increased hours of soccer per week and years of soccer experience. According to the researchers, the results suggest that even subconcussive impacts could be associated with cognitive function changes that are consistent with mild traumatic brain injury of the frontal lobes. However, further studies are needed to evaluate soccer players for longer periods to investigate if these changes are transient or longer lasting.

Although some studies have suggested some degree of association between heading and brain function impairment, others have not corroborated this result. An early study by Tysvaer and Storli (39), which addressed the effects of long-term heading exposure, aimed to examine to what extent heading produced discomfort or permanent head trouble. Male professional soccer players, aged between 18 and 34 years, answered a questionnaire elaborated to record the incidence of head injuries due to heading. None of the players had been operated on for epi- or subdural hematoma or other brain damage an only a few had suffered concussion. The results also showed that 50% of players reported acute symptoms (e.g., disorientation), 16.4% related protracted symptoms (e.g., headache), and only 4.7% described prolonged symptoms (e.g., weakened memory) due to heading. According to the authors, the questionnaire’s data suggest that there seems to be a low percentage of serious head injuries associated with heading practice, although they acknowledge that heading can be dangerous and attention should be paid to teach young players how to head correctly.

Putukian et al. (40) investigated the consequences of immediate heading exposure, by assessing cognitive function of male and female college soccer players before and after typical training sessions. The neuropsychological battery measured reaction time, concentration, attention span, speed of information processing, divided attention, and active problem solving. In one session, the athletes participated in heading practices and, in the other session, they abstained from heading during the exercises. The results showed no differences in pre-test or in post-test scores between heading and non-heading groups. A practice effect was found, since there was an increase in post-test scores compared with pre-test ones, which was consistent between groups. There were also significant differences between males and females in some cognitive test variables. As argued by the researchers, this study was exploratory in nature and only examined the acute effect of heading on a limited number of neuropsychological tests and with a limited sample.

In a study involving a younger population, Janda et al. (12) evaluated the effect of repetitive head impacts due to heading in male and female amateur soccer players with a mean age of 11.5 years, by using a neuropsychological testing protocol and documentation of concussive symptoms. The players were followed over a period of three seasons during the first year and a subgroup of male players was followed for an additional year. Thus, a short-term heading exposure was investigated in this study. The number of times a player headed the ball was monitored throughout the seasons. When comparing pre-season with post-season testing scores, the authors found no significant difference. Also, there was no evidence of difference between the scores in this study and the standardized norms. The data showed no significant correlation between the number of ball impacts and cognitive performance, with the exception of a weak inverse association involving verbal learning in the second year. Of note, however, is the fact that, in the first year, 49% of the players complained of headaches after heading the ball. As pointed out by the authors, it is unclear whether the reported headaches were consequence of mild head injuries or rather localized pain in the region of the impact.

Stephens et al. (41) compared neuropsychological test scores of male school team soccer players with those of male rugby players and non-contact sport players, all aged between 13 and 16 years. Cumulative head injury incidence and cumulative heading, an approximate calculation of long-term heading exposure, were estimated by using self-reports and a combination of observation and self-reports, respectively. The results showed no significant difference between groups in scores of 13 neuropsychological tests. Also, there was no relationship between either cumulative head injury or cumulative heading and cognitive functioning. The only exception was a marginal prediction of *TAP – Divided Attention* accuracy scores by cumulative heading, consistent with the study hypothesis of poorer neuropsychological test performance with increasing cumulative heading. According to the researchers, although this variable should be considered in further research, an interpretation of no heading effects in these adolescent soccer players is more appropriate until results of confirmatory analyses are known.

Another study, conducted by Straume-Naesheim et al. (42), examined the association between long-term heading exposure and previous concussions with performance on neuropsychological tests among male professional soccer players with a mean age of 25.6 years. The athletes completed a questionnaire assessing heading exposure and previous concussions, and a subgroup of players were observed in two to four matches for direct counting of heading actions. All participants were submitted to the computer-based neuropsychological test battery *CogSport* (43), which measures motor function, decision-making, simple, divided and complex attention, working memory, and learning and memory. The results showed no association between estimated match or lifetime heading exposure and cognitive performance. Importantly, self-reported number of headings correlated well with the observed values. Only 1.5% of the players qualified as outliers for one or more subtasks when compared with the normal range. The number of previous concussions was positively associated with lifetime heading exposure, but there was no association between previous concussions and cognitive performance. Although this study has some important limitations, it reveals no evidence of cognitive impairment caused by subconcussive and concussive trauma in soccer.

Kaminski et al. (44) investigated whether there was a relationship between number of headings taken in a season, which characterizes a short-term heading exposure, and scores on cognitive function and balance in female high-school and college soccer players (mean age 15.1 and 19.1 years, respectively). The study also involved an age- and gender-matched college control group, whose members were not participating in any organized sport. Prior to and immediately following the soccer season, all participants were given a battery of neuropsychological and postural stability tests. Heading data were documented by counting the number of times each player headed the ball during sanctioned games. In both the college and high-school groups, there were no significant correlations between the total number of headings and the change in scores of all outcome measures from pre-season to post-season. Moreover, the authors found no significant differences between the three groups in post-season scores of the neuropsychological tests, which measured concentration and immediate memory recall and verbal memory. The only significant difference was noted in post-season balance scores between the college players and the other two groups, as the first group had a worse performance. However, while the high-school and control groups were slightly improved from pre-season to post-season, the college group did not change, which suggests that one season of soccer participation did not have a detrimental effect on postural control. Therefore, overall, the results showed no evidence of cognitive or balance deficits in female soccer players.

In a further study, also involving female high-school soccer players and exploring the effects of short-term heading exposure, Kaminski et al. (45) evaluated computerized neuropsychological test performance before and after a competitive soccer season and measured the number of headings per match. There was no relationship between purposeful heading and neuropsychological performance. The results indicated that none of the cognitive measures deteriorated. Interestingly, two measures showed small but significant improvements over baseline, which the authors attribute to a test–retest practice effect. Although this investigation involved a large number of participants and provided a perspective over one playing season, studies involving longer time periods are needed to help answer the question about the potential long-term effects of heading practice.

Aiming to compare the effects of low, moderate, and high short-term heading exposure, Kontos et al. (46) investigated male and female amateur soccer players, aged between 13 and 18 years, on computerized cognitive performance and symptoms. Participants completed the *Immediate Postconcussion Assessment and Cognitive Testing* (*ImPACT*), a previously validated test battery used to assess and manage concussions in sports (47), which includes a report of concussive symptoms and measures verbal memory, visual memory, motor processing speed, reaction time, and impulse control. The researchers recorded observed numbers of headings for each player during two randomly selected practices and games for each of the soccer teams during a season. The results showed no differences in computerized cognitive performance or symptoms among low, moderate, and high heading exposure groups. Moreover, the sample of soccer players scored significantly higher across all cognitive tasks and reported fewer symptoms than the age- and gender-matched 10th percentile (i.e., unusually low) norms. A comparison between genders revealed that males headed the ball more frequently and showed lower verbal and visual memory and motor processing speed scores than females. According to the researchers, the findings do not support a relationship between soccer heading exposure and cognitive impairment and symptoms in male and female youth soccer players. They suggest that if any association exists, it is subtle and may affect only a small number of athletes, which deserves future investigation.

In a recent study, Vann Jones et al. (48) questioned whether long-term heading exposure was associated with persistent cognitive decline. Male retired professional soccer players were required to complete a self-assessed test of cognition, the *Test Your Memory* questionnaire, a previously validated tool (49). Further information was collected in order to analyze the potential effect of a number of variables on cognition. The mean age of the participants and the mean length of the professional playing career were 67.4 and 13.8 years, respectively. The results showed that 10.9% of the responders scored positively for possible mild cognitive impairment or dementia. There was no association between low-risk and high-risk playing positions, respectively, associated with reduced and greater frequency of heading, as well as length of playing career, and a positive screening result. As expected, age was a risk factor, although this was not significantly different from the local population prevalence for mild cognitive impairment across age groups over 65 years (50). Therefore, the results demonstrated no evidence of association between chronic subconcussive head injury in soccer and accelerated cognitive decline. The authors suggest that the short-term and medium-term cognitive impairment caused by heading may only be transient. Thus, once the players end their playing careers, their risk of cognitive decline would fall in line with the population. However, future longitudinal studies involving larger samples of professional soccer players are needed to support these findings.

## Final Comments

The research about the effects of heading on brain structure and function has produced intriguing results, but the findings are still inconclusive. There are very few studies involving neuroimaging techniques in order to investigate possible associations between heading practice and brain structure abnormalities in soccer players. Also, a small number of studies have evaluated biochemical markers of brain injury in these individuals. Both neuroimaging and biomarker technologies are promising areas of research that should be more explored in future investigations for assessing the effects of heading.

Although the number of studies addressing the effects of subconcussive impacts on brain function is relatively greater, when compared to the amount of investigations focused on brain structure, questions persist as to whether or not heading is deleterious to cognitive functioning. Many of these studies have methodological limitations, which should be taken into account when considering discrepancies in results, including lack of a suitable control group, failure to control for history of concussion, lack of screening for alcohol use, estimates of heading exposure based on self-reports, small sample size, low or unknown response rates, inappropriate statistical methods, among others. It is also important to note that most research in this area has concentrated on male soccer players. The growth of the female soccer population at all levels of competition (44) calls attention to the need of including these athletes in further studies.

It is noteworthy, although not focused in this review, that some investigations [e.g., see Ref. (51–55)] have addressed the effects of heading practice specifically on postural stability and oculomotor control, which ultimately also reflect brain functioning. However, their results are still inconclusive. Moreover, other relevant studies [e.g., see Ref. (56–60)] have investigated biomechanical aspects of heading, by exploring measurements of head impact, which can help to better understand the risk and safety of heading a soccer ball.

It has been estimated that professional soccer players play approximately 300 games and head the ball more than 2000 times during their careers (39). The technique of heading is complex and varies for different game situations. Proper heading technique, which involves stabilization of the neck musculature as well as the torso to reduce rotational forces, may protect soccer players from possible deleterious effects (5). In this sense, younger players may be at a great risk for head and neck injury from heading, since their technique is not yet fully developed. While learning this skill, several impacts will probably occur using an improper technique. In addition, a study involving female high-school soccer players (61) demonstrated significant, albeit moderate, negative correlations between neck strength and head acceleration from heading. Thus, as pointed out by the authors, the results suggest that athletes with weaker necks cannot tolerate headings as well as athletes with stronger necks. Therefore, more studies involving individuals in the formative years of development of soccer skills, including investigations about validity of protection equipment as well as age-appropriated soccer balls, could be valuable.

Considering that soccer extends its reach throughout the world, and it is currently the most played sport, the long-term consequences of soccer-related concussive and subconcussive brain trauma may represent a major public health problem. As emphasized by some authors (62), although a spectrum of chronic neurological injuries may occur, a primary concern involves chronic traumatic encephalopathy. All reported neuropathologically confirmed cases of this neurodegenerative disease have history of repetitive brain trauma, although not all individuals with such a history develop the pathology (63, 64). The prevalence of chronic traumatic encephalopathy, originally reported in boxers by Martland (65), is still unknown and an *in vivo* diagnosis is not currently possible, which may enhance the concern about potential consequences of heading practice.

Further studies, especially with longitudinal designs, are needed to clarify the clinical significance of heading as a cause of brain injury, which remains controversial and unexplored, and to identify risk factors. Such investigations might contribute to the establishment of safety guidelines that could help to minimize the risk of possible adverse effects of soccer on brain structure and function.

## Author Contributions

All authors gave substantial contributions to conception and design of the article. AR drafted the work, and RL and PC revised it critically. All authors approved the final version of the review and are accountable for all aspects of the study.

## Conflict of Interest Statement

The authors declare that the research was conducted in the absence of any commercial or financial relationships that could be construed as a potential conflict of interest.
